# A Paper-Based Device for Ultrasensitive, Colorimetric Phosphate Detection in Seawater

**DOI:** 10.3390/s20102766

**Published:** 2020-05-12

**Authors:** Joan M. Racicot, Teresa L. Mako, Alexander Olivelli, Mindy Levine

**Affiliations:** 1 Department of Chemistry, University of Rhode Island, 140 Flagg Road, Kingston, RI 02881, USA; joan_racicot@my.uri.edu (J.M.R.); tmako17@my.uri.edu (T.L.M.); aolivelli@my.uri.edu (A.O.); 2 Department of Chemical Sciences, Ariel University, 65 Ramat HaGolan St, Ariel 40700, Israel

**Keywords:** phosphate detection, paper-based sensors, reagent stabilization, colorimetric detection

## Abstract

High concentrations of certain nutrients, including phosphate, are known to lead to undesired algal growth and low dissolved oxygen levels, creating deadly conditions for organisms in marine ecosystems. The rapid and robust detection of these nutrients using a colorimetric, paper-based system that can be applied on-site is of high interest to individuals monitoring marine environments and others affected by marine ecosystem health. Several techniques for detecting phosphate have been reported previously, yet these techniques often suffer from high detection limits, reagent instability, and the need of the user to handle toxic reagents. In order to develop improved phosphate detection methods, the commonly used molybdenum blue reagents were incorporated into a paper-based, colorimetric detection system. This system benefited from improved stabilization of the molybdenum blue reagent as well as minimal user contact with toxic reagents. The colorimetric readout from the paper-based devices was analyzed and quantified using RGB analyses (via ImageJ), and resulted in the detection of phosphate at detection limits between 1.3 and 2.8 ppm in various aqueous media, including real seawater.

## 1. Introduction

The detection of phosphate in complex environments has been an active area of research since the 1960s, after researchers identified phosphate as a critical nutrient in eutrophication [1]. In eutrophication, phosphate and other nutrients cause toxic algae to multiply and spread uncontrollably, covering the surface of the affected water supply [2] This phenomenon is environmentally detrimental for marine and terrestrial ecosystems [3,4], and is accelerated by the widespread usage of fertilizer and phosphate-based pesticides combined with other sources of pollution including improper human waste treatment that raise the levels of environmental phosphate [5]. Regulators have attempted to address the issue of phosphate-induced accelerated eutrophication by setting low concentration limits of 0.2 and 10 ppm for phosphate in natural water and wastewater, respectively [6]. The ability to measure phosphate rapidly and effectively at these concentration limits and in complex aqueous environments is needed.

In addition to the detrimental effects of phosphate on the environment, phosphate has known deleterious effects on human health, including impaired renal function and harmful vascular calcification [7]. To minimize negative phosphate-induced effects, the World Health Organization set the maximum concentration of phosphate in drinking water at 1 ppm [8]. In order to effectively detect these low phosphate concentrations, there is a need to develop rapid, robust, and portable detection methods for phosphate that can be applied on-site. 

One commonly used method for phosphate detection in aqueous environments is to monitor color changes of a molybdenum blue-based assay [9]. In this assay, a molybdenum (IV) reagent reacts with phosphate in acidic solution to generate molybdophosphoric acid, which is subsequently reduced to generate a bright blue molybdenum-phosphate complex. Several solution-state and solid-state systems based on this method have been previously reported, yet challenges arise from the toxicity [10] and instability of the molybdenum reagent as well as its poor sensitivity (high detection limit) and poor selectivity (propensity to react with other, non-phosphate species to provide a blue readout signal) [11]. In contrast to colorimetric detection using molybdenum blue, instrumentation-based techniques have also been reported, and include the use of fluorescence spectroscopy [12], electrochemistry [13], and Raman spectroscopy [14]. While such techniques demonstrate high levels of selectivity and sensitivity, their broad-based applicability, especially for on-site measurements in complex environments, has not yet been established. Moreover, colorimetric detection has significant advantages compared to other potential methods, including ease of use [15] and the low cost of measuring color changes [16], that make it particularly attractive for phosphate detection in complex environments.

Reported herein is the development of a paper-based colorimetric phosphate detection system designed to address gaps in existing phosphate detection technology, through the development of a molybdenum-based method that limits toxic exposure to molybdenum and improves system stability, sensitivity, and selectivity. This system relies on the unique coordination ability of ethylene glycol as an additive to coordinate with and stabilize the active molybdenum complex. Although ethylene glycol has been used as a ligand for a number of metals [17], including molybdenum [18], its use as a stabilizer in colorimetric detection has not been reported to date. Of note, the ethylene glycol-stabilized molybdenum complex remained stable when adsorbed on a solid cellulose support for 30 weeks longer than an otherwise identical sample that lacked the ethylene glycol, with the use of such cellulose supports having additional advantages in terms of low toxicity [19], low cost [20], and ease of use. The newly developed system reported herein has numerous other advantages, including the ability to use a small sample volume (as little as 25 μL) and the ability to detect phosphate in ultrapure water at concentrations as low as 0.16 ppm. Comparisons of device performance at several different temperature and atmospheric humidity conditions established high levels of general applicability in real-world environments. Furthermore, excellent stability of the reagents was demonstrated, with no decrease in performance up to 250 days, when stored in dark at temperatures below 4 °C. Overall, markedly improved performance in phosphate detection was demonstrated, especially compared to current, commercially available phosphate detection methods, with significant potential for the development of improved practical phosphate detection devices.

## 2. Materials and Methods

All chemicals were purchased from Sigma-Aldrich chemical company or Fisher Scientific chemical company and used as received. Cellulose products were purchased from Fisher Scientific and residual phosphate was removed from the paper by washing it with 1.0 M HCl (three times) and ultrapure water (three times) after wax printing but prior to reagent addition. Synthetic freshwater was prepared following EPA standard procedures [21]. Synthetic seawater at a salinity of 30.5 ppt was prepared using Red Sea Coral Pro Salt mix by dissolving 33.4 g of the salt mix in 1 L ultrapure water. Water from the Sargasso Sea, a region with known low nutrient content [22], was filtered through a 0.2 µm filter to remove organic matter prior to use, as the presence of residual particulate matter has been shown to affect the accuracy and precision of colorimetric detection schemes [23]. Of note, the use of a simple syringe filter for this step means that in-field applications will be able to use similar filters without compromising widespread applicability of this method [24]. 

Dimensions for both the devices and the laminate were designed using Adobe Illustrator. Wax printing was accomplished using a Xerox Color Qube 8580 wax printer (Dedham, MA, USA), and the laminate (Fellowes 3mil self-adhesive laminate sheets) was cut using a Graphtec CE6000-40 cutting plotter. Images of device responses were collected in RAW-format using an iPhone 4 (Apply) in regular camera mode with no flash and no HDR, and the lighting of the device during image capture was controlled using a homemade lightbox. To create the lightbox, a cardboard box with an aperture cut in the top to enable cell phone-based photography was spray-painted using Krylon Fusion Satin Black spray paint (purchased from The Home Depot Warwick, Rhode, USA). LEDMO 6000K, 2835 SMD, LED white light tape was secured to the inside of the box for uniform illumination. For stability studies, images were captured using an Epson V19 Perfection flatbed scanner. Images were then processed to obtain Red Values using ImageJ software (free download from: https://imagej.nih.gov/ij/) on an 8-bit color scale (white = 255 a.u., black = 0 a.u.). The Red Values were then subtracted by 255 to provide increasing trends for color development based on concentration.

### 2.1. Reagent Preparation

The colorimetric detection method involves two reagent solutions: an ascorbic acid solution (used as a reducing agent) and an acidic mixture of molybdenum (used as the active species) and antimony (used as the co-catalyst). Both reagent solutions are commonly used for the detection of phosphate and their composition has been thoroughly optimized in previous work [11,25]. The “molybdenum reagent” was prepared as a solution of 0.126 M ammonium molybdate tetrahydrate and 6 mM potassium antimony tartrate hydrate in 6.6 M sulfuric acid, through adding the reagents and immediately mixing to achieve a homogeneous solution. This solution was diluted with ethylene glycol (to a final concentration of 4.7 M with respect to sulfuric acid) by adding 1.4 mL of ethylene glycol per 1.0 mL of initial solution. The “ascorbic acid reagent” was prepared as a 1.0 M solution of ascorbic acid in ultrapure water. All solutions were made through adding the reagents and immediately mixing them to achieve homogeneity.

The phosphate solutions were prepared from a stock solution of 1000 mg/mL of sodium dihydrogen phosphate in ultrapure water and lower concentrations of 0.1, 0.2, 0.25, 0.3, 0.4, 0.5, 0.6, 0.7, 0.8, 0.9, 1.0, 2.5, 10, and 25 ppm were obtained through serial dilution of the stock solution. 

### 2.2. Device Preparation

**Figure 1:**
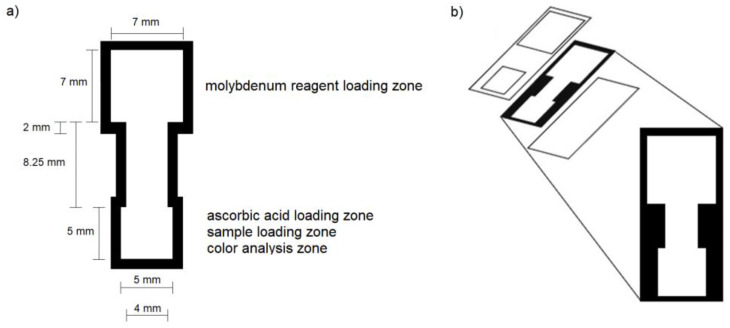
(**a**) Dimensions of the wax-printed paper device; (**b**) expanded view of device paper layer and associated laminate layers.

The paper-based devices were patterned using a wax printer onto Whatman grade 4 filter paper, according to the dimensions shown in Figure 1a. These hydrophobic wax barriers were fixed in place by melting the wax in an oven at a temperature of 120 °C for 2 min. Self-adhesive laminate sheets were placed onto the front of the devices as shown in Figure 1 so that the loading zones remained uncovered. Uncut pieces of laminate were used to cover the backs of the devices, and the laminate was sealed using pressure lamination. Whatman grade 4 filter paper had a thickness of 205 μm microns and Fellowes self-adhesive laminate sheet had a thickness of 3 mil (76 microns), making the final thickness of the device approximately 357 microns.

The final paper-based devices contained two zones: an ascorbic acid loading zone and molybdenum reagent loading zone (Figure 1). The device was designed in this way to ensure that the two reagents remained fully separated prior to device usage, as combining the two reagents led to undesired reactivity and degradation in less than 24 h [26]. Ascorbic acid was added to the devices in four separate 3 μL aliquots, with the stepwise addition used to ensure that the reagent remained in the loading zone. The devices were allowed to dry for at least 20 min between each ascorbic acid addition and prior to use of the device. No more than four aliquots could be used as excess reagent led to over-acidification of the reaction and a subsequent decrease in color readout.

### 2.3. Device Application and Color Analysis

A total of 75 μL of molybdenum reagent was added to the device via micropipette immediately prior to sample addition and allowed to flow to the ascorbic acid zone. A yellow color was observed when both reagents were allowed to mix, and 25 µL of phosphate sample was then applied to the device in the sample loading zone and allowed to develop for 4 min before image capture with a cell phone using the settings detailed above.

### 2.4. Stability Studies

The stability of these devices over time was examined by drying both the molybdenum and ascorbic acid reagent solutions on the devices. The devices were stored in sealed vials and kept in the following conditions: “light”—under ambient lighting and temperature in open air; “dry”—under ambient lighting and temperature with a *Dry and Dry* silica desiccant packet; “dark”—under ambient temperature conditions in darkness; “fridge”—at ≤4 °C in darkness; “freezer”—at ≤−18 °C in darkness. At various time points, samples were scanned with a flatbed scanner and RGB values were obtained using ImageJ software. The degradation of reagents was determined based on the formation of a blue color (indicating the degradation of the molybdenum reagent) or yellow color (indicating the degradation of the ascorbic acid reagent).

### 2.5. Limits of Detection and Quantitation

The devices were prepared as discussed in Section 2.2, and each sample point of the calibration curves was tested via three independent experiments to ensure reliability and precision. Solutions of sodium dihydrogen phosphate at concentrations of 0.0, 0.1, 0.2, 0.3, 0.4, 0.5, 0.6, 0.7, 0.8, 0.9, and 1.0 ppm were prepared via serial dilution of concentrated stock solutions made in ultrapure water, synthetic freshwater, synthetic seawater, and Sargasso seawater. Then, 25 µL of the sample solution was added to each device and the color was allowed to develop for 4 min before image capture with a cell phone. The red values were obtained using ImageJ software and OriginPro nonlinear curve fitting models were applied to the data until the best fitting line (i.e., highest R^2^ value) was obtained. Limits of detection (LOD) and limits of quantitation (LOQ) were calculated using the following equations [27]:

y_LOD_ = ȳ_B_ − 3σ_B_ (1)

y_LOQ_ = ȳ_B_ − 10σ_B_ (2)

where y_LOD_ and y_LOQ_ are the signal responses (Red Values) corresponding to LOD and LOQ values, ȳ_B_ represents the average Red Value of the blank (i.e., 0 ppm phosphate) measurement, and σ_B_ represents the standard deviation of the blank measurement. The y_LOD_ and y_LOQ_ values were substituted into the obtained nonlinear best fit equations and Excel Solver (plug-in to Microsoft Excel) was used to solve for the LOD and LOQ concentrations.

### 2.6. Environmental Robustness Studies

To simulate temperature and humidity ranges, the devices were acclimated at the desired conditions for 30 min prior to use. The temperature was controlled in a Boekel Scientific Digital Incubator and relative humidity was adjusted using Dry and Dry silica desiccant packets or water as necessary until the desired relative humidity was reached. Temperature and relative humidity were monitored using an AcuRite Digital Humidity and Temperature Comfort Monitor. Once acclimation was complete, the phosphate sample was added to the devices and the devices were returned to the incubator and the color was allowed to develop for 4 min before images were collected.

To simulate turbidity conditions, suspensions of 1, 5 and 10 mg/mL of Kaolin clay [28] in phosphate sample solutions (0, 0.5, 2.5, 5 ppm) were created, then allowed to stir vigorously overnight. 

## 3. Results and Discussion

### 3.1. Optimization of Device Parameters

**Figure 2:**
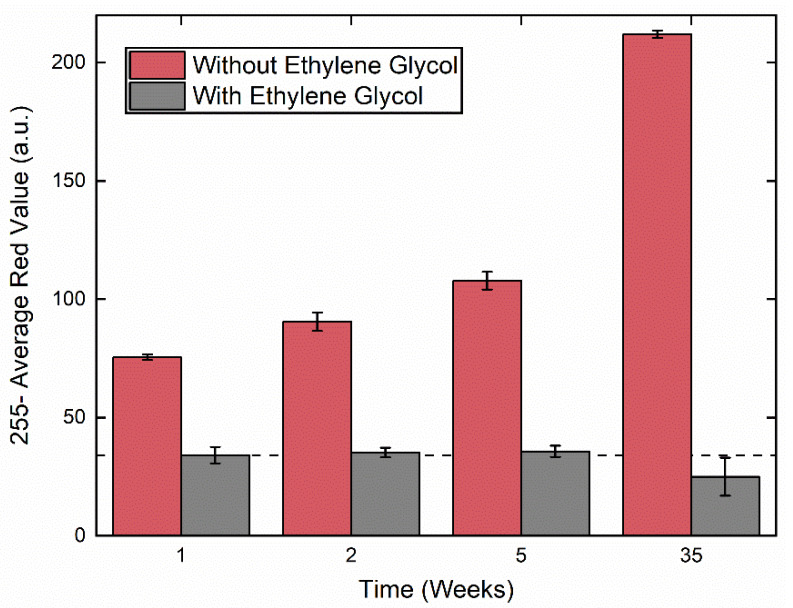
Stability studies for molybdenum blue reagent with ethylene glycol (grey bars) and without ethylene glycol (red bars) when dried on paper and stored in a refrigerator at ≤4 °C for up to 35 weeks. Stability is indicated by lack of color variation over time. The grey dashed line indicates the color of the reagent when initially dried on paper at t = 0 weeks. The error bars represent the standard deviation of three measurements.

The primary method by which the commercially available and state-of-the-art molybdenum-blue based methods for phosphate detection can be improved is by enhancing the stability of the molybdenum reagent, which has been shown to produce a blue color even in the absence of phosphate due to degradation under ambient conditions [29]. To that end, a variety of potential stabilizers for the molybdenum reagent were screened, with ethylene glycol providing the maximum reagent stabilization (see ESI for more details about the screening studies). As shown in Figure 2, the molybdenum reagent resisted degradation in the presence of ethylene glycol for up to 35 weeks when dried on paper and stored in a fridge at ≤4 °C. In comparison, under the same conditions, the completely unstabilized molybdenum reagent began to degrade within 1 week, as shown by the formation of a blue color. Additionally, the degradation rate of the ethylene glycol-stabilized reagents at room temperature was substantially slower than in the absence of ethylene glycol (Figure S7). Storage in freezer conditions (≤−18 °C) provided similar stability to fridge conditions (≤4 °C) (Figure S8), and so refrigeration was chosen for the optimal storage conditions.

The fact that ethylene glycol stabilizes molybdenum complexes has been previously reported in the literature [30], and is likely due to chelation between the small molecule diol and molybdenum that retards undesired side reactivity. Of note, other aliphatic alcohols, including glycerol and 3-propane diol, were less effective at stabilizing the molybdenum reagent, as were supramolecular additives with multiple hydroxyl moieties, including α, β, and γ-cyclodextrin. Investigations into the reasons for the unique effects of ethylene glycol are currently underway in our laboratory. 

**Figure 3:**
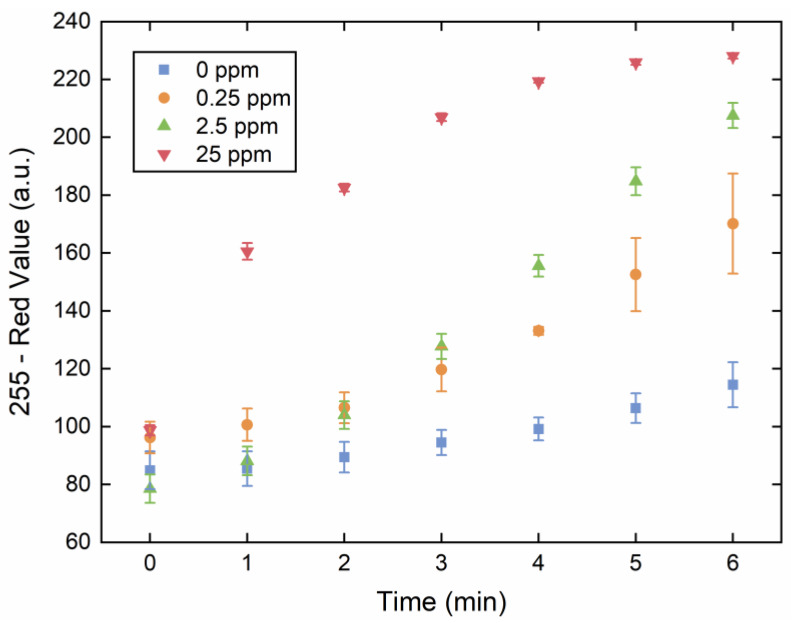
Optimization of color development (measured as 255-a.u.) over time (measured in min) to determine optimal reaction time. Sensor images were collected at 1-min intervals for devices treated with 0 ppm phosphate (blue data points), 0.25 ppm phosphate (orange data points), 2.5 ppm phosphate (green data points), and 25 ppm phosphate (red data points). The observed red values were obtained using ImageJ software for analysis. The error bars represent the standard deviation of three measurements.

In order to optimize the reaction time and color development of the device, the rate of blue colored complex formation at room temperature was examined by collecting images of the device every minute for eight minutes (Figure 3). Notably, because the rate of color formation is highly dependent on phosphate concentration, the chosen reaction time for the device must encompass the range of concentration-specific responses. Color development in higher concentrations of phosphate (25 ppm) started to slow at approximately 3 min, whereas samples with lower concentrations of phosphate (0.25–2.5 ppm) were still consistently developing color even at 8 min, although an increase in the observed standard deviation of the measurements for these mid-range concentrations was evident after 5 min. Additionally, a gradual increase in the color intensity of the blank sample (with 0 ppm phosphate) was observed due to the slow reaction of the molybdenum and ascorbic acid reagents to produce the blue color. Overall, the 4-min reaction time point was chosen for subsequent measurements because significant differences in red value between the concentrations were observed and the measurement standard deviations were still low. 

### 3.2. Detection and Quantitation Limits

**Figure 4:**
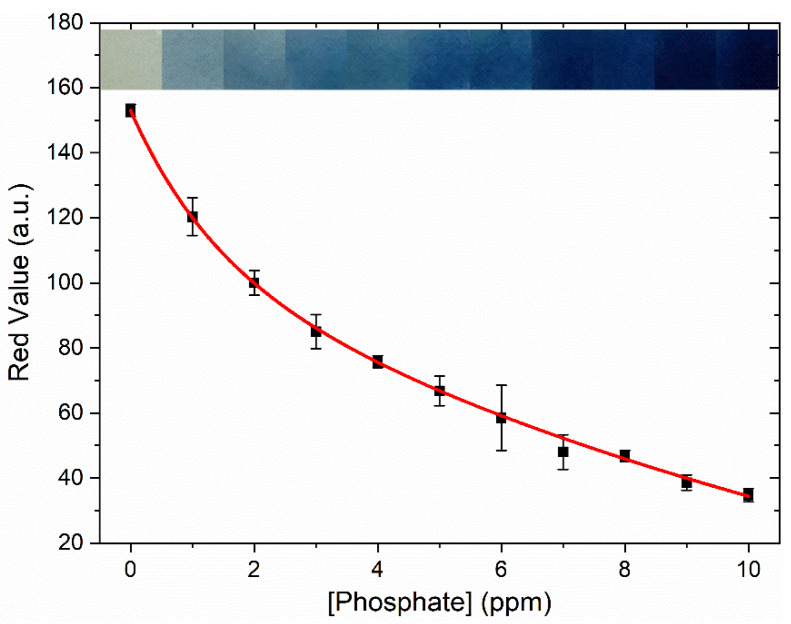
Sensor calibration curve and color gradient for phosphate concentrations of 1–10 ppm in synthetic freshwater. The data were fit to a second order exponential decay of the formula y = A_1_^(-x/t1)^ + A_2_^(-x/t2)^ + y_0;_ where A_1_ = 44.0; t_1_ = 1.34; A_2_ = 158.; t_2_ = 15.7; y_0_ = −49.0; R_2_ = 0.999. The error bars represent the standard deviation of three measurements.

In addition to demonstrating good device stability and ease of use, the ability to detect phosphate at extremely low concentrations (i.e., ppb) is critical for monitoring and controlling algal blooms. To this end, the limit of detection (LOD) and limit of quantification (LOQ) for the optimized device in ultrapure water were found to be 0.16 and 0.56 ppm, respectively (Table 1). The limits of detection and quantification were similarly low in several different aqueous media conditions, including synthetic seawater, synthetic freshwater, and a real-water sample from the Sargasso Sea, suggesting that overall salinity and trace ion content does not noticeably hinder device performance. One example of the calibration curves used to calculate LODs and LOQs is shown in Figure 4, for synthetic freshwater, with the color gradient insert showing clear color differences at the single ppm phosphate concentrations investigated. Of note, both the detection limits and working range (0.1–10 ppm) for the optimized device are lower than previously reported paper-based devices (0.62–30.7 ppm [11], 0.30–30.7 ppm [25]). Moreover, the second-order exponential decay used to fit the data suggests information about the kinetics of the reaction that underlie the blue color development [31], although detailed information about reaction kinetics on cellulose support has not yet been reported.

### 3.3. General Device Applicability

As the optimized devices require the addition of the molybdenum reagent immediately prior to sample addition, the general applicability of the device would be limited if the user had to handle toxic molybdenum reagents. To address this concern, we devised a reusable three-dimensional (3-D) printed housing for the paper device. This housing, which contains a port for the molybdenum reagent, was used with the molybdenum reagent stored in a pre-filled syringe. To use the device together with its optimized housing, the individual operator can deposit the molybdenum reagent by pressing the syringe, waiting a short time for the initial yellow color to form, adding the desired sample, and then waiting 4 min for the color to develop. The resulting color can then be imaged and analyzed using a personal cell phone device. Overall, this modification effectively minimizes safety concerns regarding the handling of toxic chemicals by the user, and eliminates potential user error from improper measuring of reagents.

**Figure 5:**
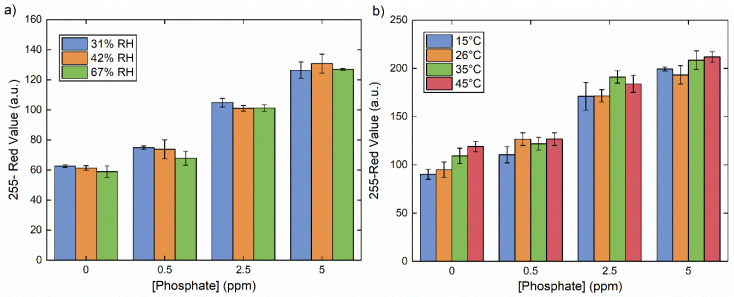
Comparison of colorimetric sensor readouts for phosphate concentrations between 0 and 5 ppm in a range of (**a**) temperature and (**b**) relative humidity (RH) conditions. The error bars represent the standard deviation of three measurements.

More general applicability considerations relate to the ability of these devices to operate at a variety of temperature and humidity conditions. Of note, previous literature-reported studies have shown that temperature has a pronounced effect on the reaction time and overall sensitivity of the molybdenum blue method [32], while humidity can have a significant effect on sample flow rate in paper-based platforms [33]. For our device, humidity values between 31% and 67% led to no discernable differences in color development for concentrations of phosphate between 0 and 5 ppm (Figure 5a). Temperature, by contrast, changed the initial color of the phosphate-free device somewhat (Figure 5b, 0 ppm phosphate), although the observed temperature effects decreased in the presence of phosphate at 0.5 ppm or above. The effects of sample turbidity were also examined through the addition of Kaolin clay to mimic natural turbidity, and the results indicated minor turbidity effects at low phosphate concentrations (≤0.5 ppm). At higher concentrations (2.5–5.0 ppm), no change was evident (Figure S16). It is possible that this is related to low levels of phosphate contamination in the clay, or to the proclivity of the reagents to also react with silica [32]. Overall, the optimized sensors are robust under most environmental conditions, with minimal effects due to temperature fluctuations and particulate content observed at extremely low phosphate concentrations. 

## 4. Conclusions

Overall, reported herein is the development of a markedly improved paper-based sensor for phosphate based on the reaction of phosphate with molybdenum blue reagents. The sensor has a number of features that enable high performance, including stabilization of the molybdenum by ethylene glycol and the use of a 3D-printed housing, that overall provide a device that can be readily used for on-site phosphate detection. The optimized device has phosphate detection limits of 0.16, 0.23, and 0.13 ppm in ultrapure water, synthetic seawater, and synthetic freshwater, respectively, significantly lower working ranges than other reported paper-based devices, and displays little to no interference over a range of temperature, humidity, and turbidity conditions. Additionally, minimal temperature effects are observed below 40 °C, although users are cautioned that using the device above 40 °C can lead to false positive results. Efforts to achieve even more robust performance and higher sensitivity are currently in progress, as are efforts to develop a practical working device, and results of these and other investigations will be reported in due course.

## Figures and Tables

**Table 1:** Limits of detection (LODs) and limits of quantitation (LOQs) in different environmental media.

Media	LOD (ppm)	LOQ (ppm)
Ultrapure water	0.16	0.56
Synthetic freshwater	0.13	0.46
Synthetic seawater	0.23	0.82
Sargasso seawater	0.28	0.99

## Supplementary Materials

The following are available online at https://www.mdpi.com/1424-8220/20/10/2766/s1, **Figure S1.** (a) Dimensions of the wax-printed paper device; (b) expanded view of device paper layer and associated laminate layers; **Figure S2.** Illustration of the colorimetric responses of Whatman #1, Whatman #4, and Whatman #41 functionalized papers to the presence of phosphate, by measuring changes in the value of 255-red value after exposure of the functionalized paper to phosphate anion; **Figure S3.** Illustration of the effects of polyol additives on the stability of the molybdenum reagent used for phosphate detection, measured as a function of the average red value (a.u.); **Figure S4.** Illustration of how different molar ratios of ethylene glycol (relative to the molybdenum complex) result in changes in the stability of the molybdenum reagent, with higher average red values (a.u.) indicating higher stability. Ratios of ethylene glycol were measured at: 0, 1, 10, 50, 75, and 100 molar equivalents, and the results represent an average of at least three trials; **Figure S5.** Summary data on the effects of exposure time on the colorimetric response of the device to phosphate concentrations of 0, 0.25, 2.5, and 25 ppm. Results were calculated using image processing software (ImageJ) and represent an average of at least three trials; **Figure S6.** Summary of the stability studies of the optimized device in the presence of ethylene glycol as an additive, measured through changes in the average red value of the functionalized paper device; **Figure S7.** Summary of the stability studies of the optimized device in the absence of ethylene glycol as an additive, measured through changes in the average red value of the functionalized paper device; **Figure S8.** Summary of stability studies of the optimized device when stored in the refrigerator with and without ethylene glycol as a stabilizing agent; **Figure S9.** Summary of stability studies of the optimized device when stored in the freezer with and without ethylene glycol as a stabilizing agent; **Figure S10.** Limit of detection of phosphate in ultrapure water, with the nonlinear best fit function shown in red. Equation: y = A1*exp(-x/t1) + y0, where y0 = 22.8; A1 = 103.3; t1 = 5.2. R2 = 0.99; **Figure S11.** Limit of detection of phosphate in synthetic freshwater, with the nonlinear best fit function shown in red. Equation: y = A1(-x/t1) + A2(-x/t2) + y0; where A1 = 44.0; t1 = 1.34; A2 = 158.; t2 = 15.7; y0 = -49.0. R2 = 0.999; **Figure S12.** Limit of detection of phosphate in synthetic seawater, with the nonlinear best fit function shown in red. Equation: y = A1*exp(-x/t1) + A2*exp(-x/t2) + y0, where y0 = -661299; A1 = 58.6; t1 = 2.8; A2 = 661353.1; t2 = 216917.9; R2 = 0.999; **Figure S13.** Limit of detection of phosphate in Sargasso seawater, with the nonlinear best fit function shown in red. Equation: y = y0 + A1*exp(-(x-x0)/t1) + A2*exp(-(x-x0)/t2); y0 = 13.8; x0 = -0.00426; A1= 56.1; t1 = 4.6; A2 = 57.4; t2 = 4.6. R2 = 0.999; **Figure S14.** Coloration of the optimized device after exposure to phosphate (0, 0.5, 2.5, and 5 ppm) in the presence of various humidity values (31%, 42%, and 67%), measured by changes in the red value of the device; **Figure S15.** Coloration of the optimized device after exposure to phosphate (0, 0.5, 2.5, and 5 ppm) at various temperatures (15, 26, 35, and 45 °C), measured by changes in the red value of the device; **Figure S16.** Coloration of the optimized device after exposure to phosphate (0, 0.5, 2.5, and 5 ppm) at various turbidity values (0, 1, 5, 10 mg/mL), measured by changes in the red value of the device. **Table S1.** Quantitative changes in the color of functionalized filter papers after exposure to phosphate anion; **Table S2.** Effects of polyol additives on the stability of the molybdenum reagent used; **Table S3.** Effects of molar equivalents of ethylene glycol added on the stability of the molybdenum reagent used, measured by the average red value; **Table S4.** Effects of exposure time on the colorimetric response of the device to phosphate concentrations of 0, 0.25, 2.5, and 25 ppm; **Table S5.** Quantitative measurements of the coloration of the device in the presence of ethylene glycol when stored under ambient light (light), with a desiccant package (dry), wrapped in foil (dark), in the refrigerator (fridge), and in the freezer (freezer); **Table S6.** Quantitative measurements of the coloration of the device in the absence of ethylene glycol when stored under ambient light (light), with a desiccant package (dry), wrapped in foil (dark), in the refrigerator (fridge), and in the freezer (freezer); **Table S7.** Quantitative measurements of the coloration of the device when stored in the refrigerator with and without ethylene glycol as a stabilizing agent; **Table S8.** Quantitative measurements of the coloration of the device when stored in the freezer with and without ethylene glycol as a stabilizing agent; **Table S9.** Summary of limit of detection and limit of quantification values of phosphate obtained using the optimized device in a variety of aqueous media; **Table S10.** Quantitative values for coloration of the optimized device at various concentrations of phosphate in the presence of variable humidity values; **Table S11.** Quantitative values for coloration of the optimized device at various concentrations of phosphate at a variety of temperatures; **Table S12.** Quantitative values for coloration of the optimized device at various concentrations of phosphate at a variety of turbidity values.
