# Supplementary material for: A Paper-Based Device for Ultrasensitive, Colorimetric Phosphate Detection in Seawater
Source: Sensors (Basel). 2020 May 12;20(10):2766. doi: 10.3390/s20102766 (PMC7294414; doi:10.3390/s20102766)
Supplement: Supplementary file 1 [file sensors-20-02766-s001.pdf]

Electronic Supporting Information for

A Paper-Based Device for Ultrasensitive, Colorimetric Phosphate Detection in Seawater

Joan M. Racicot, Teresa L. Mako, Alexander Olivelli, and Mindy Levine

TABLE OF CONTENTS

Materials and Methods……………………………………………………………………………….S3
Experimental Procedures………………………………………………………………………………S4
Optimization of Device Parameters……………………………………………………………….....S7
Summary of Device Stability Studies………………………………………………………………S10
Limit of Detection Summary Data……………………………………………………………………S13
Environmental Stability Studies………………………………………………………………………S15

MATERIALS AND METHODS

All chemicals were purchased from Sigma-Aldrich chemical company or Fisher Scientific chemical company and used as received. Cellulose products were purchased from Fisher Scientific and residual phosphate was removed from the paper by washing it with 1.0 M HCl (3 times) and ultrapure water (3 times) after wax printing but prior to reagent addition. Synthetic freshwater was prepared following EPA standard procedures. Synthetic seawater at a salinity of 30.5 ppt was prepared using Red Sea Coral Pro Salt mix by dissolving 33.4 g of the salt mix in 1 L ultrapure water. Water from the Sargasso Sea, a region with known low nutrient content, was filtered through a 0.2 µm filter to remove organic matter prior to use.

EXPERIMENTAL PROCEDURES

Experimental Procedure for Device Design

Dimensions for both the devices and the laminate were designed using Adobe Illustrator. Wax printing was accomplished using a Xerox Color Qube 8580 wax printer, and the laminate (Fellowes 3mil self-adhesive laminate sheets) was cut using a Graphtec CE6000-40 cutting plotter. Images of device responses were collected in RAW-format using an iPhone 4 (Apply) in regular camera mode with no flash and no HDR, and the lighting of the device during image capture was controlled using a homemade lightbox. To create the lightbox, a cardboard box with an aperture cut in the top to enable cell phone-based photography was spray-painted using Krylon Fusion Satin Black spray paint (purchased from The Home Depot). LEDMO 6000K, 2835 SMD, LED white light tape was secured to the inside of the box for uniform illumination. For stability studies, images were captured using an Epson V19 Perfection flatbed scanner.

Experimental Procedure for Image Processing

Images were then processed to obtain Red Values using ImageJ software (free download from: https://imagej.nih.gov/ij/) on an 8-bit color scale (white = 255 a.u., black = 0 a.u.). The Red Values were then subtracted by 255 to provide increasing trends for color development based on concentration.

Experimental Procedure for Reagent Preparation

The colorimetric detection method involves two reagent solutions: an ascorbic acid solution (used as a reducing agent) and an acidic mixture of molybdenum (used as the active species) and antimony (used as the co-catalyst). The "molybdenum reagent" was prepared as a solution of 0.126 M ammonium molybdate tetrahydrate and 6 mM potassium antimony tartrate hydrate in 6.6 M sulfuric acid. This solution was diluted with ethylene glycol (to a final concentration of 4.7 M with respect to sulfuric acid) by adding 1.4 mL of ethylene glycol per 1.0 mL of initial solution. The "ascorbic acid reagent" was prepared as a 1.0 M solution of ascorbic acid in ultrapure water.

The phosphate solutions were prepared from a stock solution of 1000 mg/mL of sodium dihydrogen phosphate in ultrapure water and lower concentrations of 0.1, 0.2, 0.25, 0.3, 0.4, 0.5, 0.6, 0.7, 0.8, 0.9, 1.0, 2.5, 10, and 25 ppm were obtained through serial dilution of the stock solution.

Experimental Procedure for Device Preparation

The paper-based devices were patterned using a wax printer onto Whatman grade 4 filter paper, according to the dimensions shown in Figure S1a. These hydrophobic wax barriers were fixed in place by melting the wax in an oven at a temperature of 120 ^{o}C for 2 minutes. Self-adhesive laminate sheets were placed onto the front of the devices as shown in Figure S1b so that the loading zones remained uncovered. Uncut pieces of laminate were used to cover the backs of the devices, and the laminate was sealed using pressure lamination.

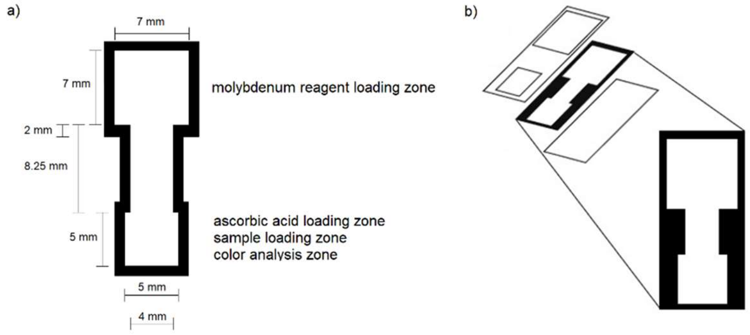

Figure S1. Dimensions of the wax-printed paper device; and b) Expanded view of device paper layer and associated laminate layers

The final paper-based devices contained two zones: an ascorbic acid loading zone and molybdenum reagent loading zone (Figure S1). The device was designed in this way to ensure that the two reagents remained fully separated prior to device usage, as combining the two reagents led to undesired reactivity and degradation in less than 24 hours. Ascorbic acid was added to the devices in four separate 3 µL aliquots, with the stepwise addition used to ensure that the reagent remained in the loading zone. The devices were allowed to dry for at least 20 minutes between each ascorbic acid addition and prior to use of the device.

Experimental Procedure for Device Application and Color Analysis

75 µL of molybdenum reagent was added to the device via micropipette immediately prior to sample addition and allowed to flow to the ascorbic acid zone. A yellow color was observed when both reagents were allowed to mix, and 25 µL of phosphate sample was then applied to the device in the sample loading zone and allowed to develop for 4 minutes before image capture with a cell phone using the settings detailed above.

Experimental Procedure for Stability Studies

The stability of these devices over time was examined by drying both the molybdenum and ascorbic acid reagent solutions on the devices. The devices were stored in sealed vials and kept in the following conditions: "light" – under ambient lighting and temperature in open air; "dry" – under ambient lighting and temperature with a *Dry & Dry* silica desiccant packet; "dark" – under ambient temperature conditions in darkness; "fridge" – at ≤ 4 ºC in darkness; and "freezer" – at ≤ -18 ºC in darkness. At various time points, samples were scanned with a flatbed scanner and RGB values were obtained using ImageJ software. The degradation of reagents was determined based on the formation of a blue color (indicating the degradation of the molybdenum reagent) or yellow color (indicating the degradation of the ascorbic acid reagent).

Experimental Procedure for Limit of Detection and Limit of Quantification Analysis

The devices were prepared as discussed above, and each sample point of the calibration curves was tested via three independent experiments to ensure reliability and precision. Solutions of sodium dihydrogen phosphate at concentrations of 0.0, 0.1, 0.2, 0.3, 0.4, 0.5, 0.6, 0.7, 0.8, 0.9, and 1.0 ppm were prepared via serial dilution of concentrated stock solutions made in ultrapure water, synthetic freshwater, synthetic seawater, and Sargasso seawater. 25 µL of the sample solution was added to each device and the color was allowed to develop for 4 minutes before image capture with a cell phone. The red values were obtained using ImageJ software and OriginPro nonlinear curve fitting models were applied to the data until the best fitting line (i.e. highest R^2 value) was obtained. Limits of detection (LOD) and limits of quantitation (LOQ) were calculated using the following equations:

$$y_{LOD} = \bar{y}_B - 3\sigma_B \quad \text{(Equation S1)}$$

$$y_{LOQ} = \bar{y}_B - 10\sigma_B \quad \text{(Equation S2)}$$

where y_{LOD} and y_{LOQ} are the signal responses (Red Values) corresponding to LOD and LOQ values, $\bar{y}_B$ represents the average Red Value of the blank (i.e. 0 ppm phosphate) measurement and σ_B represents the standard deviation of the blank measurement. The y_{LOD} and y_{LOQ} values were substituted into the obtained nonlinear best fit equations and Excel Solver (plug-in to Microsoft Excel) was used to solve for the LOD and LOQ concentrations.

Experimental Procedures for Environmental Robustness Studies

To simulate temperature and humidity ranges, the devices were acclimated at the desired conditions for 30 minutes prior to use. The temperature was controlled in a Boekel Scientific Digital Incubator and relative humidity was adjusted using Dry & Dry silica desiccant packets or water as necessary until the desired relative humidity was reached. Temperature and relative humidity were monitored using an AcuRite Digital Humidity and Temperature Comfort Monitor. Once acclimation was complete, the phosphate sample was

added to the devices and the devices were returned to the incubator and the color was allowed to develop for 4 minutes before images were collected.

To simulate turbidity conditions, suspensions of 1 mg/mL, 5 mg/mL, and 10 mg/mL of Kaolin clay in phosphate sample solutions (0, 0.5, 2.5, 5 ppm) were created, then allowed to stir vigorously overnight.

OPTIMIZATION OF DEVICE PARAMETERS

Optimization of Paper Type

A variety of filter paper types were studied for colorimetric responses to the presence of phosphate, and the results are summarized in Figure S2 and Table S1, below:

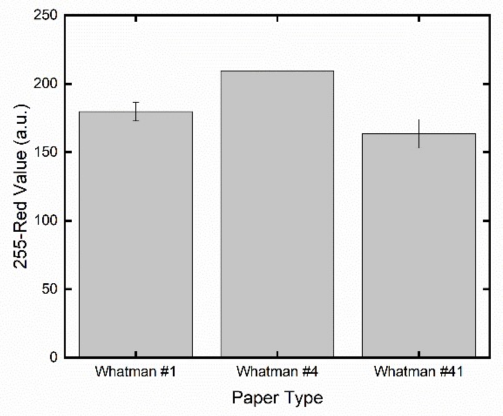

Figure S2. Illustration of the colorimetric responses of Whatman #1, Whatman #4, and Whatman #41 functionalized papers to the presence of phosphate, by measuring changes in the value of 255-red value after exposure of the functionalized paper to phosphate anion

Table S1. Quantitative changes in the color of functionalized filter papers after exposure to phosphate anion[a]

Filter paper type	Colorimetric response measured as 255-red value (a.u.)
Whatman #1	179.7 ± 6.8
Whatman #4	209.6 ± 5.7
Whatman #41	163.6 ± 10.4

[a] All results represent an average of at least three trials

Optimization of Additives

A variety of polyol additives were tested for their ability to stabilize the molybdenum reagent, and the results are summarized in Figure S3 and Table S2, below:

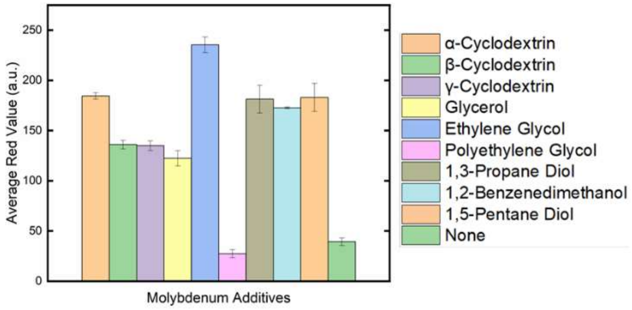

Figure S3. Illustration of the effects of polyol additives on the stability of the molybdenum reagent used for phosphate detection, measured as a function of the average red value (a.u.)

Table S2. Effects of polyol additives on the stability of the molybdenum reagent used[a]

Additive	Average red value (a.u.)

None	39.3 ± 3.8
α-Cyclodextrin	184.5 ± 3.2
β-Cyclodextrin	136.0 ± 4.3
γ-Cyclodextrin	135.0 ± 4.8
Glycerol	122.3 ± 7.6
Ethylene glycol	235.5 ± 7.8
Polyethylene glycol	27.4 ± 3.9
1,3-Propane diol	181.3 ± 13.8
1,2-Benzenedimethanol	172.6 ± 0.8
1,5-Pentane diol	183.1 ± 14.0

a All results represent an average of at least three trials

Optimization of Ethylene Glycol Ratio

A variety of ratios of ethylene glycol to the active molybdenum reagent were tested, with the goal of identifying the ratio that enabled maximum reagent stabilization. The results of these studies are summarized in Figure S4 and Table S3, below:

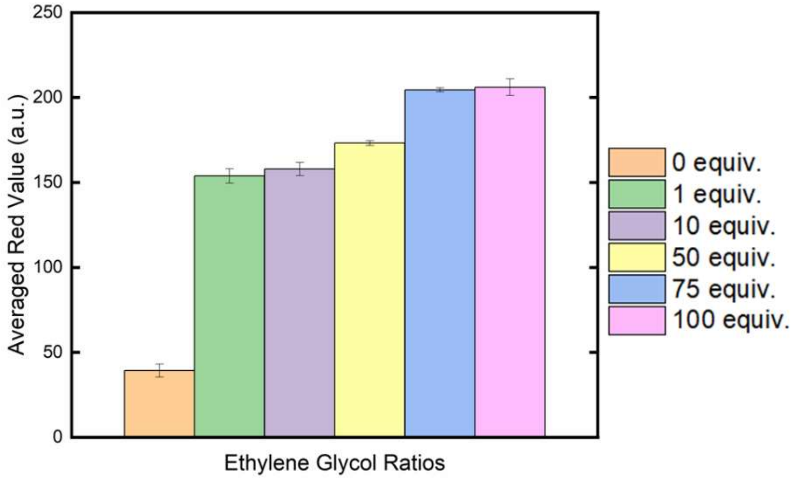

Figure S4. Illustration of how different molar ratios of ethylene glycol (relative to the molybdenum complex) result in changes in the stability of the molybdenum reagent, with higher average red values (a.u.) indicating higher stability. Ratios of ethylene glycol were measured at: 0 molar equivalents, 1 molar equivalent, 10 molar equivalents, 50 molar equivalents, 75 molar equivalents, and 100 molar equivalents, and the results represent an average of at least three trials.

Table S3. Effects of molar equivalents of ethylene glycol added on the stability of the molybdenum reagent used, measured by the average red value[a]

**Equivalents of ethylene glycol**	**Average red value (a.u.)**
0	39.3 ± 3.8
1	153.8 ± 4.2
10	157.9 ± 3.9

50	173.1 ± 1.4
75	204.5 ± 1.2
100	206.0 ± 4.9

a All results represent an average of at least three trials

Optimization of Exposure Time to Phosphate

The amount of time to expose the functionalized device to phosphate was measured, using phosphate concentrations of 0 ppm, 0.25 ppm, 2.5 ppm, and 25 ppm, and the coloration of the device after exposure to these concentrations was measured in one-minute intervals up to six minutes. The results of these optimization trials are summarized in Figure S5 and Table S4, below:

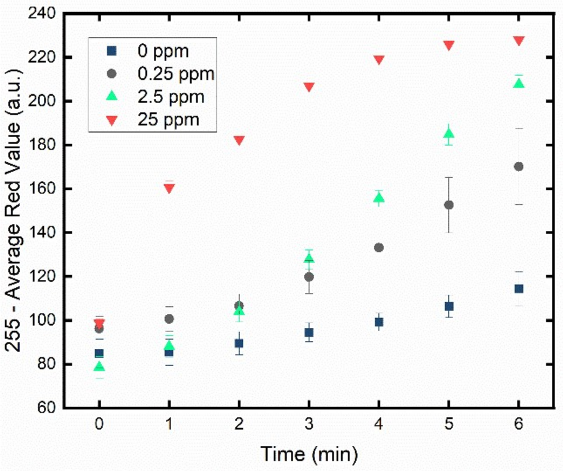

Figure S5. Summary data on the effects of exposure time on the colorimetric response of the device to phosphate concentrations of 0 ppm, 0.25 ppm, 2.5 ppm, and 25 ppm. Results were calculated using image processing software (ImageJ) and represent an average of at least three trials.

Table S4. Effects of exposure time on the colorimetric response of the device to phosphate concentrations of 0 ppm, 0.25 ppm, 2.5 ppm, and 25 ppm[a]

Exposure time (minutes)	Colorimetric response (255-average red value) (a.u.)			
	**0 ppm phosphate**	**0.25 ppm phosphate**	**2.5 ppm phosphate**	**25 ppm phosphate**
0	84.9 ± 6.5	96.2 ± 5.4	78.5 ± 4.9	98.8 ± 1.8
1	85.4 ± 6.0	100.6 ± 5.6	88.1 ± 4.9	160.6 ± 2.9
2	89.5 ± 5.3	106.5 ± 5.3	104.0 ± 4.8	182.6 ± 1.3
3	94.5 ± 4.3	119.7 ± 7.6	127.8 ± 4.3	206.9 ± 1.3
4	99.2 ± 4.0	133.1 ± 1.4	155.6 ± 3.7	219.4 ± 0.5
5	106.4 ± 5.1	152.6 ± 12.7	184.8 ± 4.8	225.9 ± 0.9
6	114.4 ± 7.8	170.1 ± 17.3	207.5 ± 4.4	228.1 ± 0.9

a All results represent an average of at least three trials.

SUMMARY OF DEVICE STABILITY STUDIES

The stability of the optimized device was measured under a variety of conditions, including temperature variations, exposure to/ protection from light, and with or without protection from ambient moisture. The results of these studies are summarized in the graphs and tables shown below:

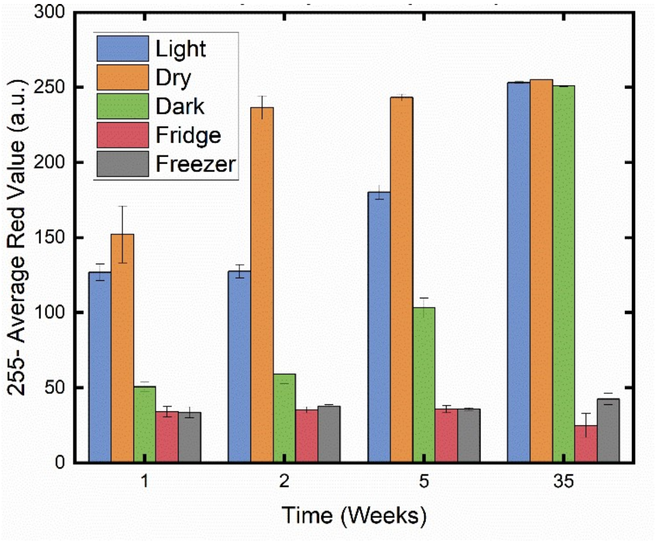

Figure S6. Summary of the stability studies of the optimized device in the presence of ethylene glycol as an additive, measured through changes in the average red value of the functionalized paper device.

Table S5. Quantitative measurements of the coloration of the device in the presence of ethylene glycol when stored under ambient light (light), with a desiccant package (dry), wrapped in foil (dark), in the refrigerator (fridge), and in the freezer (freezer)[a]

Time (weeks)	Colorimetric response (255-average red value) (a.u.)				
	Light	Dry	Dark	Fridge	Freezer
1	126.9 ± 5.6	152.1 ± 18.9	50.6 ± 3.4	34.1 ± 3.5	33.7 ± 3.5
2	127.5 ± 4.4	236.5 ± 7.8	59.2 ± 6.6	35.3 ± 2.0	37.8 ± 1.0
5	180.3 ± 4.8	243.2 ± 2.3	103.2 ± 6.5	35.7 ± 2.4	35.7 ± 0.9
35	253.2 ± 0.6	255.0 ± 0.0	250.9 ± 0.4	25.0 ± 8.0	42.6 ± 3.8

[a] All values represent an average of at least three trials and were obtained using ImageJ software for data analysis and quantification.

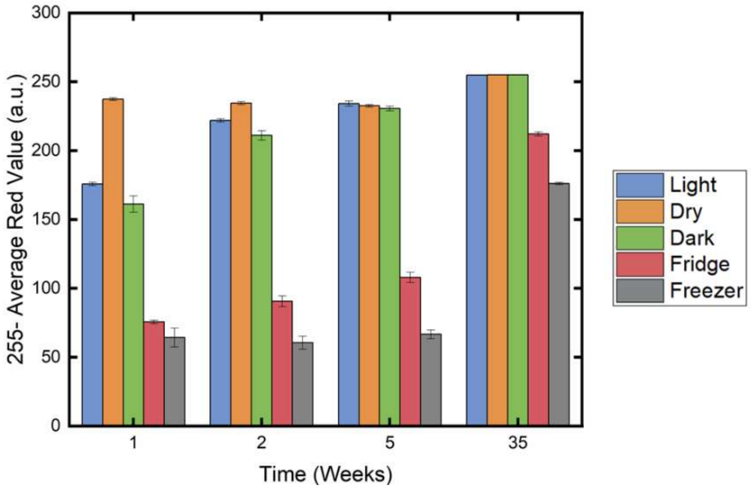

Figure S7. Summary of the stability studies of the optimized device in the absence of ethylene glycol as an additive, measured through changes in the average red value of the functionalized paper device.

Table S6. Quantitative measurements of the coloration of the device in the absence of ethylene glycol when stored under ambient light (light), with a desiccant package (dry), wrapped in foil (dark), in the refrigerator (fridge), and in the freezer (freezer)[a]

Time (weeks)	Colorimetric response (255-average red value) (a.u.)				
	Light	Dry	Dark	Fridge	Freezer
1	175.8 ± 1.2	237.3 ± 1.0	161.2 ± 5.9	75.6 ± 1.2	64.2 ± 6.9
2	221.9 ± 1.2	234.5 ± 1.2	211.1 ± 3.4	90.5 ± 3.9	60.5 ± 4.6
5	234.1 ± 1.9	232.5 ± 0.9	230.6 ± 1.7	107.9 ± 3.8	66.6 ± 3.1
35	254.9 ± 0.0	255.0 ± 0.0	255.0 ± 0.1	212.1 ± 1.6	176.1 ± 0.8

[a] All values represent an average of at least three trials and were obtained using ImageJ software for data analysis and quantification.

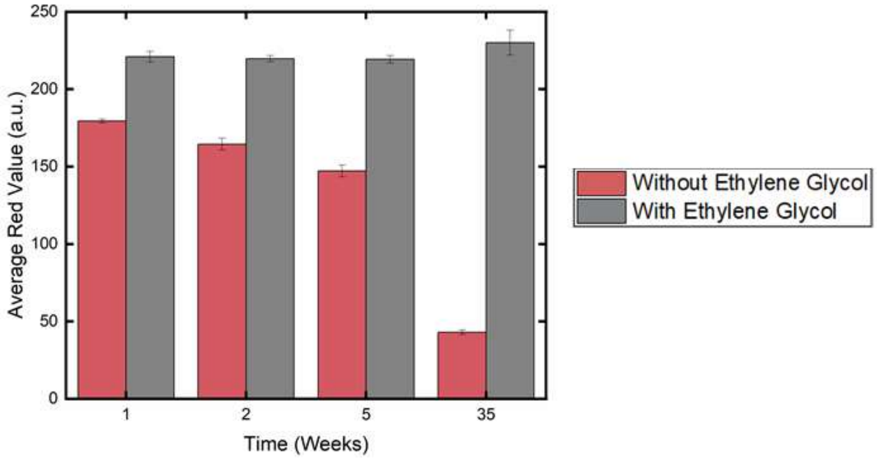

Figure S8. Summary of stability studies of the optimized device when stored in the refrigerator with and without ethylene glycol as a stabilizing agent

Table S7. Quantitative measurements of the coloration of the device when stored in the refrigerator with and without ethylene glycol as a stabilizing agent[a]

Time (weeks)	Average Red Value (a.u.) without Ethylene Glycol	Average Red Value (a.u.) with Ethylene Glycol
1	179.4 ± 1.2	220.9 ± 3.5
2	164.5 ± 3.9	219.7 ± 2.0
5	147.1 ± 3.8	219.3 ± 2.4
35	42.9 ± 1.6	230.0 ± 8.0

[a] All values represent an average of at least three trials and were obtained using ImageJ software for data analysis and quantification.

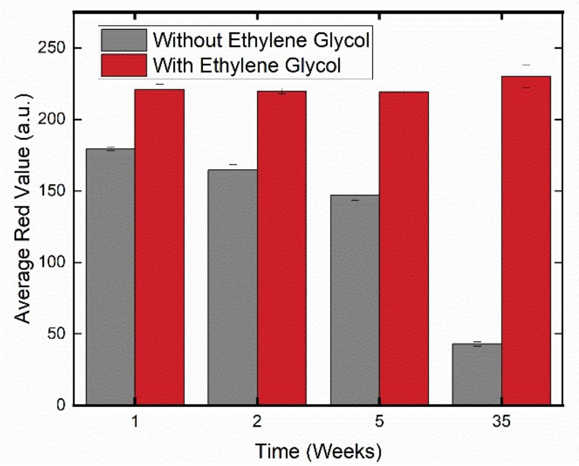

Figure S9. Summary of stability studies of the optimized device when stored in the freezer with and without ethylene glycol as a stabilizing agent

Table S8. Quantitative measurements of the coloration of the device when stored in the freezer with and without ethylene glycol as a stabilizing agent

Time (weeks)	Average Red Value (a.u.) without Ethylene Glycol	Average Red Value (a.u.) with Ethylene Glycol
1	64.2 ± 6.9	33.7 ± 3.5
2	60.5 ± 4.6	37.8 ± 1.0
5	66.6 ± 3.1	35.7 ± 0.9
35	176.1 ± 0.8	42.6 ± 3.8

[a] All values represent an average of at least three trials and were obtained using ImageJ software for data analysis and quantification.

LIMIT OF DETECTION SUMMARY DATA

Limits of detection were calculated for the optimized device in ultrapure water, synthetic freshwater, synthetic seawater, and Sargasso seawater, and results of these studies are summarized in the figures and tables below:

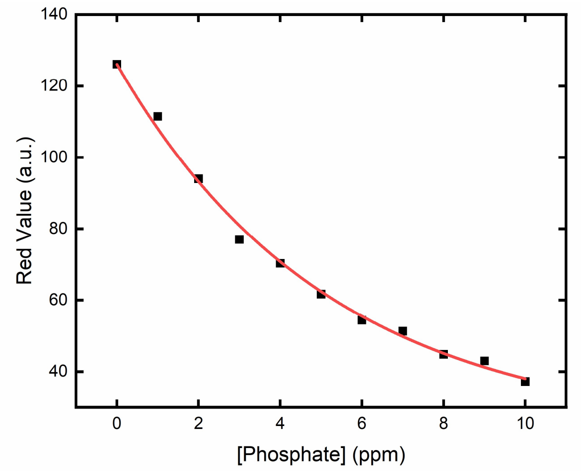

Figure S10. Limit of detection of phosphate in ultrapure water, with the non-linear best fit function shown in red. Equation: y = A1*exp(-x/t1) + y0, where y0 = 22.8; A1 = 103.3; t1 = 5.2. $R^2 = 0.99$

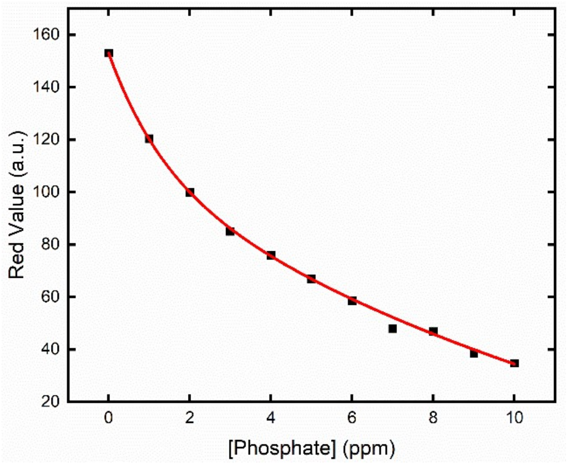

Figure S11. Limit of detection of phosphate in synthetic freshwater, with the non-linear best fit function shown in red. Equation: $y = A_1^{(-x/t1)} + A_2^{(-x/t2)} + y_0$; where $A_1 = 44.0$; $t_1 = 1.34$; $A_2 = 158.$; $t_2 = 15.7$; $y_0 = -49.0$. $R^2 = 0.999$.

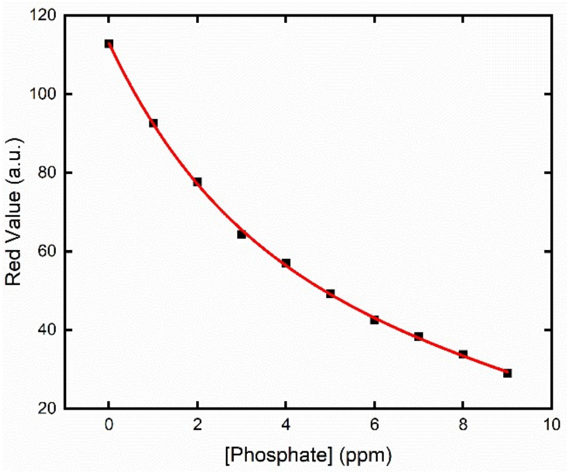

Figure S12. Limit of detection of phosphate in synthetic seawater, with the non-linear best fit function shown in red. Equation: y = A1*exp(-x/t1) + A2*exp(-x/t2) + y0, where y0 = -661299; A1 = 58.6; t1 = 2.8; A2 = 661353.1; t2 = 216917.9; R^2 = 0.999.

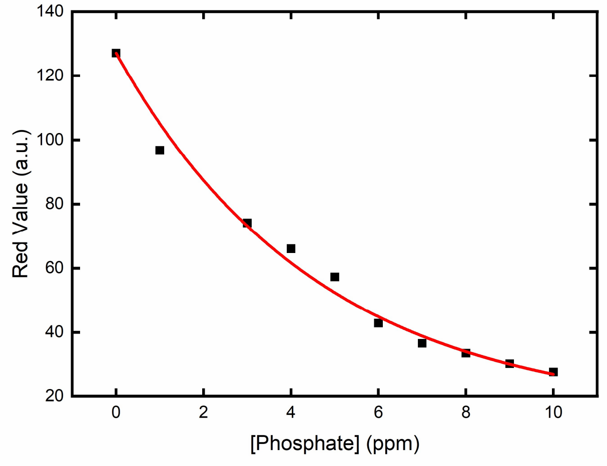

Figure S13. Limit of detection of phosphate in Sargasso seawater, with the non-linear best fit function shown in red. Equation: y = y0 + A1*exp(-(x-x0)/t1) + A2*exp(-(x-x0)/t2); y0 = 13.8; x0 = -0.00426; A1= 56.1; t1 = 4.6; A2 = 57.4; t2 = 4.6. R^2 = 0.999.

Table S9. Summary of limit of detection and limit of quantification values of phosphate obtained using the optimized device in a variety of aqueous media

Aqueous Media	Limit of Detection (ppm)	Limit of Quantification (ppm)
Ultrapure water	0.16	0.56
Synthetic freshwater	0.13	0.46
Synthetic seawater	0.23	0.82
Sargasso seawater	0.28	0.99

ENVIRONMENTAL STABILITY STUDIES

The optimized device was tested under a variety of conditions, including variable humidity values, temperatures, and turbidities, and the results are summarized in the figures and tables below.

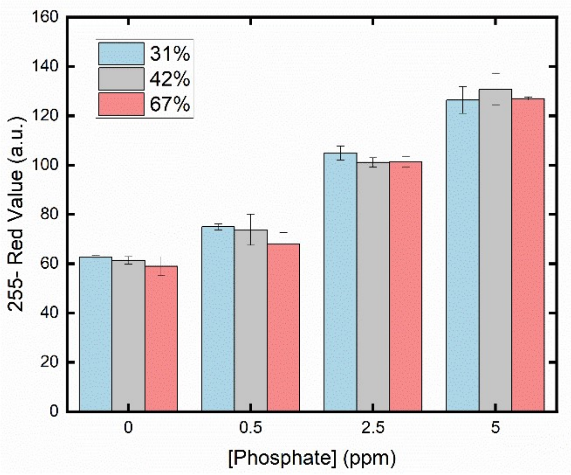

Figure S14. Coloration of the optimized device after exposure to phosphate (0 ppm, 0.5 ppm, 2.5 ppm, and 5 ppm) in the presence of various humidity values (31%, 42%, and 67%), measured by changes in the red value of the device

Table S10. Quantitative values for coloration of the optimized device at various concentrations of phosphate in the presence of variable humidity values[a]

[Phosphate] (ppm)	255-Red Value (a.u.) at 31% humidity	255-Red Value (a.u.) at 42% humidity	255-Red Value (a.u.) at 67% humidity
0	62.6 ± 0.8	61.3 ± 1.6	58.9 ± 3.8
0.5	74.9 ± 1.2	73.8 ± 6.3	67.9 ± 4.7
2.5	104.8 ± 2.9	101.1 ± 1.9	101.2 ± 2.1
5	126.3 ± 5.5	130.8 ± 6.3	126.9 ± 0.6

[a] All results represent an average of at least three trials

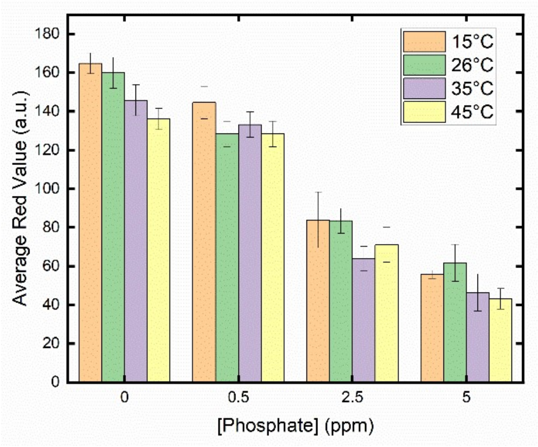

Figure S15. Coloration of the optimized device after exposure to phosphate (0 ppm, 0.5 ppm, 2.5 ppm, and 5 ppm) at various temperatures (15 °C, 26 °C, 35 °C, and 45 °C), measured by changes in the red value of the device

Table S11. Quantitative values for coloration of the optimized device at various concentrations of phosphate at a variety of temperatures[a]

[Phosphate] (ppm)	Average Red Value at 15 °C	Average Red Value at 26 °C	Average Red Value at 35 °C	Average Red Value at 45 °C
0	164.8 ± 5.3	160.0 ± 8.0	145.7 ± 8.0	136.0 ± 5.5
0.5	144.5 ± 8.4	128.3 ± 6.5	133.1 ± 6.5	128.3 ± 6.6
2.5	83.9 ± 14.4	83.5 ± 6.4	63.9 ± 6.4	71.0 ± 9.0
5	55.7 ± 2.1	61.7 ± 9.6	46.4 ± 9.6	43.1 ± 5.3

[a] All results represent an average of at least three trials

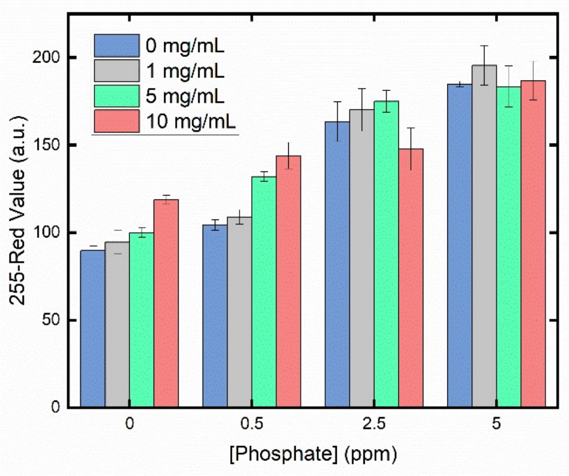

Figure S16. Coloration of the optimized device after exposure to phosphate (0 ppm, 0.5 ppm, 2.5 ppm, and 5 ppm) at various turbidity values (0 mg/mL, 1 mg/mL, 5 mg/mL, 10 mg/mL), measured by changes in the red value of the device

Table S12. Quantitative values for coloration of the optimized device at various concentrations of phosphate at a variety of turbidity values[a]

[Phosphate] (ppm)	255-Red Value at 0 mg/mL	255-Red Value at 1 mg/mL	255-Red Value at 5 mg/mL	255-Red Value at 10 mg/mL
0	89.6 ± 2.6	94.5 ± 6.8	99.9 ± 2.7	118.9 ± 2.4
0.5	104.3 ± 3.0	108.9 ± 4.1	132.0 ± 2.7	143.9 ± 7.5
2.5	163.4 ± 11.3	170.1 ± 12.1	175.0 ± 6.3	147.7 ± 12.2
5	184.8 ± 1.7	195.4 ± 11.3	183.4 ± 11.7	186.8 ± 11.1

[a] All results represent an average of at least three trials
